# Update on Fever of Unknown Origin in Children: Focus on Etiologies and Clinical Approach

**DOI:** 10.3390/children11010020

**Published:** 2023-12-24

**Authors:** Sandra Trapani, Adele Fiordelisi, Mariangela Stinco, Massimo Resti

**Affiliations:** 1 Department of Health Sciences, University of Florence, 50139 Florence, Italy; 2 Pediatric Unit, Meyer Children’s Hospital IRCCS, 50139 Florence, Italy; adele.fiordelisi@unifi.it (A.F.); massimo.resti@meyer.it (M.R.); 3 Liver Unit, Meyer Children’s Hospital IRCCS, 50139 Florence, Italy; mariangela.stinco@meyer.it

**Keywords:** fever of unknown origin, infectious, inflammatory, neoplastic, children, workup

## Abstract

Fever of unknown origin (FUO) can be caused by four etiological categories of diseases. The most common cause of FUO in children is represented by infections, followed by inflammatory conditions and neoplastic causes; a decreasing quote remains still without diagnosis. Despite the fact that several diagnostic and therapeutic approaches have been proposed since the first definition of FUO, none of them has been fully validated in pediatric populations. A focused review of the patient’s history and a thorough physical examination may offer helpful hints in suggesting a likely diagnosis. The diagnostic algorithm should proceed sequentially, and invasive testing should be performed only in select cases, possibly targeted by a diagnostic suspect. Pioneering serum biomarkers have been developed and validated; however, they are still far from becoming part of routine clinical practice. Novel noninvasive imaging techniques have shown promising diagnostic accuracy; however, their positioning in the diagnostic algorithm of pediatric FUO is still not clear. This narrative review aims to provide a synopsis of the existent literature on FUO in children, with its major causes and possible diagnostic workup, to help the clinician tackle the complex spectrum of pediatric FUO in everyday clinical practice.

## 1. Introduction

Fever of unknown origin (FUO) is a common condition worldwide in both adults and children. However, it remains a challenge for even the most experienced pediatrician, as it requires fine skills both in history and objective examination, as well as in the subsequent choice of the most appropriate diagnostic process. In addition, FUO has a significant impact on the health system and is responsible for 3% of hospitalizations [1].

Normally, most feverish diseases resolve before the origin or cause can be clarified, but sometimes fever prolongs without reaching a diagnosis [2]. As known, fever can have several patterns [3]: continuous, if the temperature remains high all day with fluctuation below 1 degree; remittent, if the temperature undergoes wide daily fluctuations (2–3 degrees), without falling <37 °C; intermittent, if hours of fever alternate with others of apyrexia on the same day; periodic, if it appears every 3–4 days; and recurrent, if the feverish episodes last a few days, interrupted by days or weeks of apyrexia [4].

When approaching a child with FUO, the first step is the demonstration of fever by healthcare personnel [1]. At the initial visit, if the fever is actually not running and the child is in good condition, the parents should be invited to return during the fever [2]. If the patient is hospitalized, observing the temperature curve helps to visualize the fever pattern. Furthermore, in suspicion of fictitious fever, it is useful to ask parents to keep a “fever diary” [3,4].

To better understand this intriguing issue, we conducted an extensive literature review of all reported pediatric cases of FUO. This review was conducted using Embase^®^, MEDLINE^®^, and MEDLINE^®^ In-Process to identify studies on FUO in childhood published as full-text articles from 1961 to October 2023. Databases were searched by combining the keywords “Fever of unknown origin” AND “child” OR “children” OR “infancy”. Articles were included in our study when matching the following eligibility criteria: (1) they provided original data on case series or case reports, and (2) the patients reported were younger than 18 years.

In 1961, Petersdorf and Beeson conducted a prospective study at Yale University on 100 adults with fever > 38.3º C for more than 3 weeks, without a diagnosis, despite a week of hospital investigations. They intended to study all unknown cases of prolonged fever, defined for the first time as “unexplained fever of origin”, based on the following criteria: temperature above 38,3ºC and longer than three weeks at home or than a week if hospitalized [5]. For years, the few studies that have focused on the specific definition of FUO in pediatrics have provided slightly different parameters for the value, frequency, and duration of fever [6]. The most appropriate current definition provides for a temperature above 38.3 °C, at least once a day, for more than 8 days in a child evaluated on an outpatient basis by the pediatrician (with inconclusive history, physical examination, and first-level investigations) [3]. In this respect, it is also useful to distinguish FUO from fever without source (FWS), based on the fever duration, which, in the latter case, is less than a week [7]. FWS may progress towards the FUO if the fever persists beyond the first week, always without cause [3,7].

## 2. Etiology

**Figure 1:**
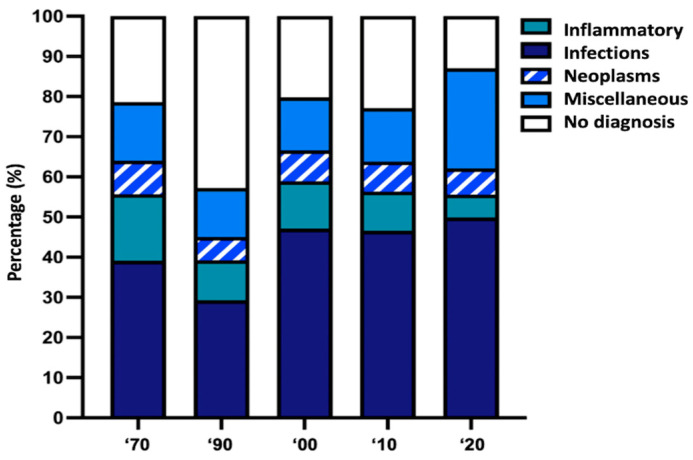
Varied distribution of the different causes of FUO over time.

Four etiological categories of diseases should be considered as causing FUO: infectious, which is the most frequent; inflammatory; neoplastic; and miscellaneous. Additionally, a variable quote remains without diagnosis even after thorough investigations. Over the years, the categories of FUO have remained the same but with different rates. The first American studies on pediatric FUO showed that, in 90% of cases, the cause was identifiable: infectious in 50%, inflammatory in 10–20%, and oncological in 10% [8,9,10]. Subsequent studies showed different results: from 20% to 44% infectious, from 0% to 7% inflammatory, from 2% to 3% oncological, and up to 67% without diagnosis [11,12]. The reason for the apparent increase in the number of cases without diagnosis in a setting of improved diagnostic techniques is unclear. The advent of improved culture techniques and the greater understanding of the pathogenesis of atypical viruses and bacteria, as well as autoimmune processes, has facilitated the early etiological diagnosis (of infectious and inflammatory forms) so that febrile children fell into the FWS group, reducing the share of infectious and inflammatory causes with consensual increase of that without diagnosis [2]. Indeed, however, the increasingly fine diagnostic techniques currently allow doctors to reach a diagnosis in an increasing number of cases. Therefore, in the last decade, the rate of children with no diagnosis has decreased, as shown in Figure 1.

Information on etiology rates derived from an analysis of the published series over the last 50 years [8,9,10,11,13,14,15,16,17,18,19,20,21,22,23,24,25,26,27] (Supplementary Table S1).

The recent literature shows that, in developing countries, the cause of FUO remains primarily infectious due to several factors: the increased prevalence of certain infections causing FUO, such as human immunodeficiency virus (HIV), tuberculosis, leishmaniasis, and malaria; the highest proportion of vaccine-preventable diseases; and reduced public health prevention’s programs, poor access to healthcare facilities, and reduced availability of diagnostic tests [28,29]. In addition, the inadequate knowledge and diagnostic capacity of some recent causes, such as hemophagocytic lymphohistiocytosis (HLH), limit the share of noninfectious forms [3].

Several differences are found in infectious causes between developing countries, where brucellosis is the main cause, followed by typhus, rickettsiosis, and tuberculosis, and developed countries where other bacterial infections, such as osteomyelitis, bartonellosis, and viral infections, particularly from Ebstein–Barr Virus (EBV) are frequent [30].

However, even in the more recent case series from the developed countries, the infectious diseases are still the most prevalent, ranging from 37.2% [25] to 50.9% [27], followed by inflammatory (from 4.9% [27] to 27,5% [24]); the neoplastic diseases represent the last (but not least) group, with rates ranging from 4.5% [25] to 17% [24].

In addition, the causes of FUO are distributed differently in diverse ages. Infections are, by far, the majority in the first year of life and decrease later, always remaining, however, the first cause. According to a recent Chinese study of 1288 cases of FUO, the share due to infectious diseases decreases with increasing age from 73% to 44%, and bacterial infections are the main cause in children below 1 year of age, while viral infections are between 1 and 6 years [27]. Neoplastic or autoimmune diseases become more frequent at later ages [22,28]. This trend is also confirmed by Yachie et al., who compared the causes of FUO in children and adulthood when autoimmune and neoplastic forms are more represented [31].

Table 1 summarizes the numerous causes of FUO, divided into infectious and noninfectious diseases, which include inflammatory diseases; neoplasia; and a last group, so-called miscellaneous, collecting rare but clinically relevant causes, such as autoimmune diseases, drug fever, diabetes insipidus, etc. A substantial share of children, variable in different case series from 10% [18] to 43% [21], however, remains undiagnosed even after an intense workup.

### 2.1. Infections

In all case series, infections account for the largest share of diagnosed cases, ranging from 19% [21] to 69% [17], including bacterial, viral, fungal, and parasite diseases. Many localized infections, including sinusitis, mastoiditis, pneumonia, abscesses, pyelonephritis, osteomyelitis, pyomyositis, infectious endocarditis (IE), and pericarditis, initially present with prolonged fever but few/no signs of localization. The IE is an example: its nuanced clinic does not immediately allow for a diagnosis; a slight heart murmur may not be perceived, and other suggestive signs of septic embolisms, such as Osler’s nodules, scleral, retinal, sub-nail hemorrhages, or on plants/palms, are rare. Osteoarticular infections that usually have peculiar symptoms can sometimes occur only with fever, especially in young children; with osteomyelitis; and if pelvic or flat bones are involved.

Intra-abdominal abscesses can also cause FUO initially without abdominal disturbances, particularly if the patient has a history of previous abdominal disease or abdominal surgery. Pyogenic hepatic abscesses usually occur in immunocompromised children but are also possible in immunocompetent ones; patients typically have hepatomegaly and tenderness in the right upper quadrant, but they sometimes have only fever. Granulomatous hepatitis, caused by various organisms, including Bartonella, is a rare cause of FUO in pediatrics [32]. Infections of the upper airways (chronic/recurrent otitis media, chronic/ recurrent pharyngitis, tonsillitis, and peritonsillar abscess), and related cavities (mastoiditis and sinusitis) are often present as FUO in children with scarce or ignored localized symptoms (cough and rhinitis). Urinary tract infection is one of the most common causes of pediatric FUO. Moreover, given the increasing number of children carrying devices, it is also worth mentioning the infection of intravascular catheters as a further cause of FUO.

Among the generalized infections giving fever without specific manifestations, we find brucellosis, caused by the ingestion of unpasteurized milk or raw/undercooked meat and characterized by recurrent fever, typically associated with profuse sweating, arthro-myalgia, and sometimes splenomegaly. Cat scratch disease, due to *Bartonella henselae* infection, is one of the most common causes of FUO and is often accompanied by lymphadenopathy and hepatosplenomegaly. In the monocentric study by Jacobs et al., *Bartonella henselae* was responsible for 5% of all FUO [12]. Latero-cervical lymphadenitis of the neck may also be due to anaerobic bacteria such as *Fusobacteriun necrophorum*, potentially responsible for bacterial thrombophlebitis of the cervical veins with associated bacteriemia, pulmonary septic embolism, and/or pneumonia (Lemierre’s disease) [33]. Salmonella sp., a possible contaminant of poultry and eggs and transmitted through contact with animal feces, can cause gastroenteritis (minor salmonellosis) and typhoid fever (*Salmonella typhi* and *S. paratyphi*). Tuberculosis (TB) is another major cause of pediatric FUO, most often in the extra-pulmonary form (disseminated TB or liver, peritoneum, pericardium, or genitourinary tract) compared to the lung. Non-tubercular mycobacteria may also cause disseminated infection, most commonly in children with HIV or other T-cell immunodeficiencies [34].

Viral infections are often responsible for FUO. Cytomegalovirus infection (CMV), usually characterized by an asymptomatic course, can cause prolonged fever in a small proportion of children who are also immunocompetent. Mononucleosis frequently causes fever and asthenia in healthy children; angina, lympho-adenomegaly, hepatomegaly, and palpebral edema may also be absent. Adenovirus infections cause feverish pharyngotonsillitis, with an increase in inflammatory indices mimicking a bacterial infection. HIV may also be responsible for fever, especially for the many opportunistic infections possible in infected children [35,36]. Recently, enterovirus has also been described as being responsible for FUO in pediatrics [37].

Concerning the geographical area, other agents, such as spirochaetes, rickettsia, and parasites, must be considered. Among the spirochetes present in our hemisphere, Lyme disease (with fever, arthralgia, and erythema marginate) supported by *Borrelia burgdoferi* should be mentioned, as well as leptospirosis, a common zoonosis recently spread not only among veterinarians, farmers, and butchers but also among children in contact with infected animals or waters, which cause myalgia, headache, cough, and gastrointestinal symptoms, in addition to fever.

Among the parasites of temperate areas, *Toxoplasma gondii*, transmitted through raw/undercooked meat, vegetables, and water contaminated by feline feces or direct contact, is responsible for toxoplasmosis that is often manifested only with fever and modest lympho-adenomegaly. Recently, there has also been an increase in cases of leishmaniasis, a disease characterized by prolonged fever, splenomegaly, and pancytopenia, due to *Leishmania donovani* and *infantum* that, through the sting of phlebotomies, passes from the animal tank to the human [38]. Malaria is to be considered in children who, after traveling in countries with high endemic disease, have FUO and splenomegaly; such a parasite can arise months after the trip and in subjects who have performed regular prophylaxis, too [39]. The last category of infectious agents is represented by fungi, which have recently been increasing: candida, aspergillus, and cryptococcus must be researched and constitute a real danger for the high risk of dissemination, especially in immunodeficient children.

### 2.2. Autoimmune Inflammatory Diseases

Among the inflammatory forms, rheumatological diseases represent a significant share, varying from 2% [17] to 4.9% [27] to 37.2% [26]; These include juvenile idiopathic arthritis (JIA), systemic lupus erythematosus (SLE), vasculitides, such as Kawasaki disease (KD), panarteritis nodosa (PAN), Behçet’s syndrome, Wegener’s granulomatosis, and antiphospholipid syndrome. In children under 16 years, a prolonged high spiking fever may suggest the systemic form of JIA, especially if associated with musculoskeletal symptoms, fleeting salmon rash, lymphadenomegaly, hepatosplenomegaly, and pleuro-pericarditis. The diagnosis of systemic JIA, which represents 15–20% of all forms of JIA, remains by exclusion. The newly proposed JIA classification states that sJIA is equivalent to adult-onset Still disease, and, therefore, the presence of arthritis is no longer mandatory [40]. In addition to fever, the presence of a classic rash plus the presence of at least two secondary criteria are sufficient for diagnosis.

SLE can start before the age of 16 with severe organ involvement, varying according to ethnicity and sex, with higher prevalence in non-European populations and females: fever, although not a diagnostic criterion, is documented in almost half of pediatric patients. A high fever (>38.5 °C) for at least 5 days, associated with at least four of the five clinical criteria (non-secretive conjunctivitis, polymorphic rash, cheilitis, extremity abnormalities, and unilateral cervical lymphadenopathy) suggests KD, vasculitis of medium/small caliber vessels typically affecting children under 5 years [41]. It should be stressed that incomplete forms in which fever accompanies only some of the above signs are more frequent in children under a year, who are at greater risk of coronary abnormalities. PAN is another vasculitis of medium-sized arteries, rare in children, diagnosed by a typical histological or arteriographic pattern of multiple aneurysms in any district, which usually begins with prolonged fever.

Wegener’s granulomatosis is a more complex vasculitis that is typically characterized by involvement of the upper respiratory tract, lungs, and kidneys, but that manifests by FUO in 22% of pediatric cases [42]. Among the inflammatory diseases, chronic inflammatory bowel diseases (IBDs) play an important role; in particular, Crohn’s disease should be considered if the child also has oral or pharyngeal aphthae, weight loss, and diarrhea [14,43].

### 2.3. Neoplastic Diseases

An important group of diseases that manifest themselves with FUO is represented by neoplasms; in some case series, these represent a small quote, ranging from 2.5% [14] to 4.5% [25], while in others, they constitute a significant percentage (16–17%), although still the minority [22,23,24]. Among these, lymphoproliferative diseases are the most frequent; however, some solid tumors, such as neuroblastoma, Wilms*’* tumor, and soft tissue sarcomas such as rhabdomyosarcoma, may also manifest as prolonged fever before determining specific signs/symptoms. Other rare neoplasms are hepatoma, pheochromocytoma, and, finally, atrial myxoma.

Other neoplastic-like forms, which, in some cases, are considered to be in the “miscellaneous” category, include histiocytosis X, HLH [44], macrophage activation syndrome, and Castleman Disease. HLH is a nonmalignant inflammatory syndrome but life-threatening, in which the uncontrolled proliferation of activated lymphocytes and histiocytes leads to hemophagocytosis and immune dysregulation with hypersecretion of inflammatory cytokines. It may be primitive, due to genetic disorders, or secondary to acquired conditions such as infection, immunological disorders, malignancy, or medication. It begins, more often in young children, as a fever but rapidly progresses towards septic-like features; its typical manifestations are prolonged fever, hepatosplenomegaly, hyperferritinemia, and cytopenia. Therefore, a high index of suspicion is needed to place such a diagnosis that, if late, can determine a poor prognosis. A useful “red flag” to suspect HLH during fever is an abrupt ESR fall [45]. Macrophage activation syndrome is a severe condition also due to a dysfunctional immune response, very similar to HFH, secondary to rheumatological diseases that can manifest with prolonged fever, splenomegaly, and pancytopenia [46]. Finally, Castleman’s disease should be mentioned, which, in its monocentric form, is a benign tumor lymphadenopathy often asymptomatic or accompanied by fever; in its most severe multicentric form, it is a cause of fever, pancytopenia, multiorgan failure, and malignancy [47].

### 2.4. Miscellaneous

In this group, disparate conditions such as drug fever, autoinflammatory diseases, hyperthyroidism, diabetes insipidus, immunodeficiency, Kikuchi–Fujimoto disease, etc., are included. Drug fever, a common condition that is often left undiagnosed, is a febrile response that coincides with the administration of a drug and disappears after its interruption. It is usually suspected when no other cause for fever can be clarified, sometimes after antimicrobial therapy has already been started. In sensitized subjects who receive a drug for the first time, the onset of fever is variable and differs between classes of drugs, but it most commonly appears after 7–10 days from the drug’s beginning and stops at its withdrawal. Early detection can reduce inadequate and potentially harmful diagnostic–therapeutic interventions; the reintegration of the offending agent usually causes the rapid resumption of fever, confirming the diagnosis, but such a practice is controversial.

A wide variety of drugs, most of them used also in children, are implicated in FUO, with different pathophysiological mechanisms; particular attention should be paid to the role of antimicrobial agents, including antibiotics and antivirals. Besides this prevalent class, pediatricians should consider several groups of drugs as being possibly responsible for FUO, such as antiepileptics (barbiturates, carbamazepine, and phenytoin), anti-inflammatory (ibuprofen, salicylates, and sulfasalazine), anticoagulants (heparin), diuretics (furosemide and hydrochlorothiazide), neuromuscular blocking agents (rocuronium and mivacurium), immunosuppressant (azathioprine, everolimus, mofetil mycophenolate, and sirolimus), and antiblastics (6-mercaptopurine, bleomycin, cisplatin, daunorubicin, and vincristine) [48].

Autoinflammatory diseases, which are relatively rare in children, have been known for about 20 years [49]. Recently, they have been defined as clinical disorders characterized by an abnormal increase in inflammation, mediated mainly by cells and molecules of the innate immune system, with a significant predisposition of the host [50]. The prototype of such forms is Familial Mediterranean Fever; however, an increasing number of conditions are ascribed to this category [31].

Such syndromes are often overlooked in the initial differential diagnosis of children with FUO; however, if the doctor considers this possibility, he/she can easily diagnose through appropriate genetic investigations because many of them have a monogenic origin [31]. Among the miscellaneous are also the rare Kikuchi–Fujimoto disease, a benign necrotizing lymphadenitis, most often caused by posterior cervical lymph nodes, mostly affecting adolescents [51]. In the pandemic, a new condition has been added to this list: the multisystem inflammatory syndrome in children (MIS-C), a rare complication of coronavirus disease 2019 (COVID-19) that presents with persistent fever associated with various cardiovascular signs/symptoms (tachycardia and murmurs) and gastrointestinal and mucocutaneous symptoms and requiring a timely diagnostic–therapeutic framework.

## 3. Clinical Approach to a Child with FUO

The evaluation of FUO should be systematic and guided by medical history and a clinical exam. An in-depth history and objective examination and interpretation of the first laboratory tests are the fundamental focus. The pace by which to proceed in subsequent evaluations and whether the patient can be managed as an outpatient or hospitalized depends on how the child looks at the first visit [3].

### 3.1. Focused History

The first step in a proper history should start with the best definition of fever in terms of duration, height, and trend. As already mentioned, the type of fever can give some suggestions about the cause. It is also useful to know how the temperature is measured and what makes it lower (for example, in KD, paracetamol is usually ineffective). The physiological history must include questions about ethnicity and genetic background, as well as the geographical area. All events that occurred in recent months, particularly in the one preceding the fever, must be investigated. First, ask about the history of travel not only in the recent past but also in the past years; this provides tips on specific infections based on geographical area.

In addition, the overall exposure to domestic and wild animals and the occurrence of insect bites represent a risk of zoonoses, which, in recent years, have also increased in developed countries [52]. The infectious diseases transmitted by animals are numerous, and they can be divided according to the different environments in which children meet the animals; indeed, they can contract zoonosis either at home, with dogs, kittens, domestic turtles, and birds; or outside, attending farms (horses, cattle, and sheep), the woods (squirrel, ferrets, rodents, and snakes), or even rivers, lakes, and seas (fishes, oysters, and clams) [52].

The history must also investigate contact with sick people or high-risk exposures (such as travel abroad, visits to prisons, or homelessness); dietary habits and intake of new foods (such as fresh cheese, goat’s milk, and unpasteurized dairy products), well water, or meat not/not cooked deserve special attention [4], as well as the presence of pica for a possible infection with *Toxocara canis*. In adolescents, sexual habits, piercing or tattooing, and exposure to narcotic substances should be evaluated. Finally, it is necessary to report the vaccinations, including the date of the most recent one.

In addition to the comorbidities, the recurrence of infectious episodes (suggestive of immunodeficiency), the presence of medical devices (central venous catheters and/or percutaneous endoscopic gastrostomy), and recent surgical interventions must be reported. Finally, the presence of systemic symptoms such as anorexia; weight loss; night sweating; itching; arthralgia; abdominal pain; alterations of stools, such as diarrhea or melena; and the association or not with chills (present in urinary infections, abscesses, and malaria) should be investigated [36]. Every detail can be relevant; therefore, a well-structured and targeted story is essential, as well as its repetition, because the parents themselves may remember new details when first omitted [3].

Table 2 summarizes the anamnestic data and the associated possible diagnoses.

### 3.2. Physical Examination

A detailed physical examination evaluating each organ or system, to be repeated day by day, is essential to reveal alterations that may also associate with each other, providing important diagnostic suggestions [2]. The detection of vital signs related to fever is crucial, particularly the relationship between heart rate and fever height: patients with typhoid fever or other enteric fevers may have bradycardia compared with high fever. In addition, height and weight must be compared with previous measurements: a decrease in growth rate or weight loss suggests an unrecognized chronic condition; typically, the more chronic the process, the less likely the cause of FUO is to be infectious. A marked cutaneous pallor is present in lymphoproliferative diseases, as well as inflammatory and autoimmune diseases. Weight loss with a history of prolonged diarrhea and abdominal pain suggests IBD, particularly Crohn’s disease, as well as lymphoproliferative disorders. Some skin lesions may be very suggestive: a fleeting salmon-pink skin rash is characteristic of systemic JIA; a seborrheic rash may be a sign of histiocytosis X; the malar rash is typical of SLE but also Parvovirus infection; and petechiae are present in vasculitis, as well as in IE or leukemias.

Fixed lymphadenopathy, especially if supraclavicular, with hepatosplenomegaly may indicate malignancy, as well as EBV infection; on the other hand, when it is isolated, cervical, unilateral, and painful, it is more often an expression of contiguous infectious processes and Lemierre’s syndrome should be considered. More rarely, lymphadenomegaly is a finding of KD or, exceptionally, Kikuchi syndrome–Fujimoto [53].

Pharyngeal hyperemia may suggest infectious mononucleosis, CMV infection, toxoplasmosis, MK, or leptospirosis. Exudative tonsillitis with splenomegaly is present in infectious mononucleosis, adenovirus infection, or Fusobacterium necrophorum infection, especially if associated with painful neck swelling. A persistent mucopurulent nasal discharge may be a sign of sinusitis, hypertrophy or inflammation of the gums and loss of teeth may indicate a form of leukemia, and recurrent oral candidiasis can be found in immunodeficiency states. Severe pain localized to the face, especially if in the malar or frontal region, can be indicative of sinusitis or mastoiditis; if the pain is evoked by the percussion of a tooth, an underlying abscess will most likely be present; an asymmetry of the pharynx and/or the presence of torticollis suggest/s peritonsillar or parapharyngeal abscesses.

The evaluation of meningeal signs is also useful: children with mild meningisms may have subacute or chronic tuberculous or fungal meningitis; locoregional infectious foci such as mastoiditis or vertebral osteomyelitis can also cause meningeal irritation. The ocular examination, which should never be missed, can show conjunctival redness that is found in adenovirus infection, KD (bulbar sparing limbus), and leptospirosis, conjunctival petechiae indicative of IE [36]; it can also reveal abnormal movements, such as opsoclonus–myoclonus, a finder of neuroblastoma. A severe joint, bone, or muscle pain associated or not with signs of inflammation may indicate a musculoskeletal infection, as well as a neoplasm; back pain with limitation of flexion–extension can suggest discitis or spondylodiscitis, as well as neoplasia, such as sarcoma or neuroblastoma. A cardiac evaluation can reveal arrhythmias present in various febrile conditions, such as rheumatic disease, Lyme disease, and myocardial abscesses; a new heart murmur may be a revealing sign of IE; rubs and muffled tones may suggest an infection or effusion in the pericardium [4]. Finally, it is necessary to carefully look for signs of localization to reveal infectious diseases of organs or systems, such as abdominal abscesses, pyelonephritis, osteomyelitis, pyomyositis, etc., requiring immediate medical and/or surgical interventions. Certainly, the presence of some signs and symptoms, such as weight loss, night sweats, itching, asthenia, pallor, petechiae, and the deterioration of general conditions, as well as the simple refusal to play, represents alarm signals, so-called “red flags”, which require a rapid and thorough diagnostic workup [6,28]. It is useful to emphasize the need to repeat the physical examination several times to re-evaluate or appreciate new or modified clinical signs compared to the first evaluation; this repetition is essential, as up to 25% of signs appear after the first assessment [8]. Supplementary Table S2 shows the main signs and possible associated pathologies.

## 4. First-Level Investigations

### 4.1. Hematochemical Tests

**Figure 2:**
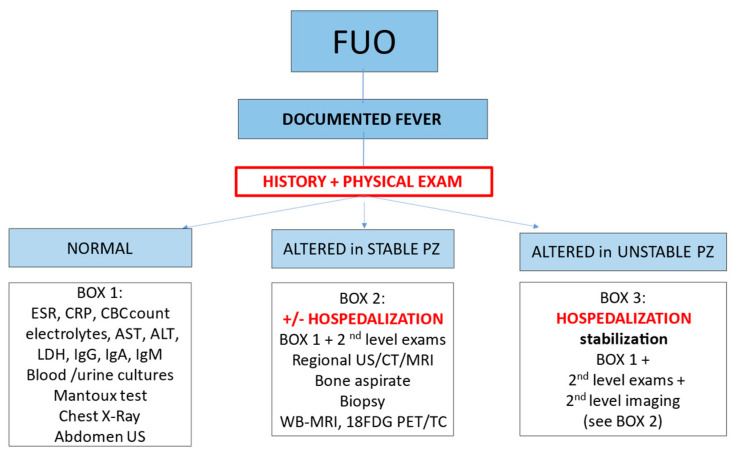
FUO algorithm in children.

The evaluation of a child with FUO cannot exclude a thorough history and careful physical examination of the patient. After the initial evaluation, the pediatrician will choose the most suitable setting to continue the investigations (outpatient or hospitalized). Figure 2 shows a proposed algorithm for the approach to the child with FUO [54]. The decision of when to hospitalize and how rapidly to proceed with invasive testing depends on multiple factors, such as the child’s general condition, the occurrence of pathological signs revealed by examination, and or significant anamnestic findings (i.e., risk exposures and comorbidities).

Children with a medical history non−evocative of any red flag and with an unremarkable physical examination (with vital parameters within normal range) may undergo only first−line blood tests (as listed in Box 1, Figure 2), along with first−level imaging tests (chest X−ray and abdominal ultrasound). In some cases, procalcitonin (PCT), immunoglobulins, ferritin, uricemia, muscular enzymes, Mantoux testing, and pharyngeal swabs may be also considered in this population.

#### 4.1.1. Complete Blood Cell Count (CBC)

The most frequently reported causes of CBC abnormalities are anemia evocative of rheumatic disorders (JIA, SLE, and KD), as well as infections (such as malaria, tuberculosis, or endocarditis), IBD, and malignancy; cytopenia of ≥1 cell lines and/or immature forms—leishmaniasis, leukemia, HLH [55], and SLE; atypical lymphocytes, which are associated with some viral infections (e.g., EBV and CMV), Kikuchi–Fujimoto disease [56], and also lymphoproliferative disorders; lymphocytosis—cat scratch disease, EBV, and toxoplasmosis; eosinophilia: parasitic or fungal infections, allergies, neoplasms, immunodeficiencies, and drug fever; thrombocytosis, which is associated with JIA, as well as KA (generally lately); and thrombocytopenia, which is found in viral infections, leptospirosis, tularemia, and malaria, as well as in autoimmune diseases, such as SLE.

#### 4.1.2. Inflammatory Biomarkers

Inflammatory markers (WBC count, ESR, CRP, and PCT) are among the most useful biochemical parameters in the evaluation of children with FUO. A significant elevation of these indices may indicate an inflammatory or infectious process, although lacking specificity. Indeed, a significant elevation in ESR and CRP decreases the likelihood of fictitious fever and prompts further investigation. On the other hand, the negativity of inflammatory biomarkers decreases the likelihood of infectious and/or inflammatory causes and, if fever is documented, leads to consider alternative and rarer diagnoses. A recent cohort study including 1288 children with FUO showed significantly higher values of WBC count, ESR, CRP, and PCT in the children affected by inflammatory diseases than in those with viral infections [22]. In contrast, previous studies did not find significant a difference in CRP among different FUO causes, whereas ESR was significantly higher in children with autoimmune and infectious diseases [23].

Regarding ESR, it is worth noting that its value can be affected by several other variables: e.g., ESR can be falsely elevated during anemia, hypergammaglobulinemia, and hyperfibrinogenemia, while it can be falsely decreased in fibrinogen-consuming diseases, such as intravascular coagulopathy and HLH [45]. Moreover, ESR tends to elevate late, even up to a week after disease onset, and it also returns slowly within normal ranges, with only limited value both in the early diagnostic phase and when assessing response to treatments.

In contrast, CRP and especially PCT are more sensitive than ESR in detecting inflammation in the earlier phases of disease. However, the clinical significance of PCT in the diagnostic workup of FUO is still unclear: the accuracy of PCT is superior to that of CRP for severe bacterial infections but tends to lose validity in the advanced phases of fever [57]. Some authors have proposed serum ferritin as a potential biomarker for the diagnostic algorithm of FUO, suggesting its usefulness in distinguishing infectious and noninfectious causes, especially when its values are considerably high. A recent study by Otrock et al. demonstrated an association between ferritin values above 10,000 μg/L and the development of HLH in children [58]. Another multicenter observational study reported that, in patients with FUO evaluated by a pediatric rheumatologist, serum ferritin levels at least five times higher than normal values proved to be a useful screening test for the diagnosis of inflammatory causes of FUO (i.e., systemic JIA). Regarding the diagnostic role of inflammatory biomarkers in the workup of children with FUO, to date, wider and ideally prospective studies are deemed to evaluate the combination of biomarkers to increase their diagnostic accuracy.

#### 4.1.3. Urine Examination

Relevant alterations in urine examination include pyuria associated with bacteriuria evocative of urinary tract infection; sterile pyuria, which is often seen in KD, intra-abdominal infection, and genito-urinary tuberculosis; hematuria and/or proteinuria, associated with SLE, as well as some infections, such as leptospirosis; and low urine-specific gravity or low osmolality associated with diabetes insipidus.

#### 4.1.4. Culture Tests

Blood cultures may provide a diagnosis in cases with infectious causes of FUO. Ideally, blood cultures should be drawn at febrile acme and, if possible, repeated over time. Blood cultures should include aerobic, anaerobic, and fungal investigations. Depending on the different clinical suspicions, cultures may also be performed from urine, feces, synovial fluid, gastric aspirate, spinal fluid, and biopsied tissues [3]. It is worth noting that, despite the fact that blood cultures are a milestone of the diagnostic workup of FUO, some evidence suggests little utility of blood cultures in modifying the empiric antibiotic therapy in children hospitalized for community-acquired infections [59].

### 4.2. Imaging

Early imaging includes chest X-rays, abdominal ultrasound (US), and echocardiogram with EKG. Numerous alterations to the chest X-ray suggest the diagnosis: i.e., infiltrated and/or thickened means pneumonia; lymphadenopathy may suggest tuberculosis or lymphoma; mediastinal mass is found in leukemia, lymphoma, neuroblastoma, or rhabdomyosarcoma; and small nodular densities, such as leptospirosis, etc. Abdominal US may detect abdominal abscesses (hepatic, subphrenic, or pelvic) or malignant masses (neuroblastoma, Wilms tumor, lymphomas, etc.), as well as signs of pyelonephritis or IBD. A complete cardiological evaluation that includes EKG and echocardiogram is mandatory in the case of a child with FUO to highlight possible cardiac involvement (pericarditis, IE, and rheumatic fever) or alterations compatible with MK (dilated coronary), especially in incomplete forms and in infants below one year of age. Children with musculoskeletal manifestations should first perform a joint/bone X-ray and then a muscle or joint US.

## 5. Second-Level Investigations

If history and physical examination provide specific clues or red flags, second-level investigations are deemed and should be selected upon diagnostic suspicion (Table 3). Hospitalization is not always mandatory for this level of investigation. Indeed, indications for hospital admission should include suboptimal general conditions or unstable clinical conditions, rapid progression of symptoms, clinical suspicion of Munchausen by proxy, the need for monitoring, and/or the need for more complex investigations/procedures [54]. It is also worth highlighting that such patients often require a multidisciplinary approach, which may be easier for inpatients [24].

### 5.1. Second-Line Laboratory Tests

The choice of second-level examinations should be guided by diagnostic hypothesis. The infectious investigations, selected upon clinical suspicion, include specific viral and bacterial serologies and viral genome searches with PCR methods. The increased incidence of TB requires the execution of QuantiFERON and/or Mantoux testing, not only in cases of active exposure to infected patients but also for patients coming from endemic regions. For such epidemiologic consideration, some authors include TB tests in first-level evaluations [60]. Similarly, malaria research should be performed in a febrile child coming from endemic regions [39]. A febrile child with lymphadenopathy, asthenia, lymphopenia, and high transaminases should be checked for mononucleosis; if the child has a history of contact with kittens, *Bartonella henselae* infection should be excluded. When immune-mediated disorders are suspected, specific tests should be chosen concerning the hypothesized disease: if JIA is suspected, titration of antinuclear antibodies (ANAs) and a slit-lamp ophthalmologic examination should be performed. If rheumatic fever is suspected, a previous streptococcal infection should be ruled out (demonstrating an increase in the ASO titer in at least two evaluations). If SLE is suspected, ANA, anti-dsDNA, and complement fractions should be measured. If the child complains of abdominal pain, associated with leukocytosis and high inflammatory markers, serodiagnosis, stool culture tests, and serology for gastrointestinal pathogens should be performed. If IBD is suspected, ASCA and ANCA should be titrated, and fecal calprotectin should be tested.

In the clinical suspicion of neuroblastoma (e.g., systemic hypertension, spinal or thoracic pain, and opsoclonus–myoclonus syndrome), urinary vanillylmandelic acid should be assayed; and, if in doubt of pheochromocytoma, urinary metanephrines should be tested. Lastly, due to several chromosomal abnormalities that have recently been recognized as potentially related to a fever of an inflammatory nature, some authors suggest that genetic sequencing, using “next-generation sequencing” methods, could be taken into consideration in selected cases [61].

### 5.2. Imaging

Second-level instrumental investigations that are also guided by the clinical suspect and district involved include Computed Tomography (CT), Magnetic Resonance Imaging (MRI), scintigraphy, endoscopy, medullary aspirate, and tissue biopsies. A CT scan with/without contrast shows different lesions in the skull, chest, abdomen, and pelvis. For example, skull CT with breast study is still considered the gold standard in suspected sinus or mastoid infection because occult sinusitis is a relatively common cause of FUO in children [4]. Similarly, in the case of a strong clinical suspicion of abdominal abscess, with negative ultrasound, a contrast-enhanced CT scan may be diagnostic for increased sensitivity. Similarly, suspecting musculoskeletal infection in which initial investigations have not provided precise framing, it is necessary to proceed with locoregional MRI. Facing febrile patients with lymphadenopathy, hepatosplenomegaly, and other severe clinical manifestations (weight loss and sweating), bone marrow aspiration, and/or lymph node biopsy might reveal lymph proliferative disorder, but also infectious diseases (viruses, bacteria, mycobacteria, Leishmania, and fungi) [62]. In the suspicion of IBD, subsequent instrumental investigations include the US, with the study of the thickness of the loops, esophagogastroduodenoscopy, and colonoscopy with related biopsies. Furthermore, if the cause of the fever remains undiagnosed after first-level imaging and/or secondary level regional imaging, then newer global imaging modalities such as whole-body MRI (WB-MRI) and/or nuclear medicine imaging are deemed [63]

Over the past two decades, several studies have pointed out the potential role of whole-body MRI (WB-MRI) in the pediatric setting. Indeed, the current indications for WB-MRI’s use in children include the investigation of FUO, avoiding ionizing radiation, analyzing areas (soft tissues) poorly evaluated upon CT, and simultaneously studying more than one district with a single sedation. A recent retrospective study, conducted on 92 children, reported data on WB-MRI use in the setting of FUO. The authors showed that such an imaging modality has a high negative predictive value, allowing doctors to discontinue the diagnostic and therapeutic workup in case of its negativity (in 2/3 of patients) [64]. Moreover, a retrospective Italian study explored the use of WB-MRI in pediatric patients with FUO and presenting clinical and laboratory pictures of ambiguous interpretation. The authors reported that WB-MRI may be useful to rule out neoplastic, as well as infectious, causes of FUO [65]. Overall, WB-MRI can be considered a good screening test for FUO with conventional negative imaging. However, as it is not specific; its positivity requires further investigation (biopsy, medullary aspirate, or sectoral MRI).

The use of scintigraphy is proposed among the investigations in the FUO also in pediatric-age patients, especially if a malignancy is assumed, after an initial inconclusive workup [66]. Recently, the ^18^Fluorodeoxyglucose (FDG) PET/CT, combining the anatomical study with the functional one, has proved the test of imaging of nuclear medicine of first choice to be superior in terms of accuracy to the CT and the previous scintigraphy with 67 Ga-citrate and marked leucocytes. Therefore, many researchers have focused on the use of this method in the diagnostic process of FUO, although pediatric studies are still few. In a study evaluating the role of FDG-PET/CT in children with FUO, the authors reported that this technique led to the identification of the cause of fever in almost half of the cases (53/110, 48%) [67]. A recent retrospective study analyzed the diagnostic power of FDG PET/CT in 35 children with FUO, showing how this method was diagnosed in all cases with only two false negatives and one false positive [68]. In addition to its application in the diagnostic process of FUO, some authors propose its use in monitoring the response to treatment; Chamroonrat et al. suggest that this method can provide prognostic information, as its negativity seems associated with a favorable outcome [63]. However, it is worth remembering that, in addition to the non-optimal diagnostic accuracy (possible false positives and negatives), FDG PET/CT exposes the patient to ionizing radiation; therefore, its use must be properly considered. So far, the application of FDG PET/TC is standardized only for entities with significant activation of the immune system, such as HLH [69].

## 6. Management and Prognosis

The appropriate management of the child with FUO cannot refrain from clear communication with the family about the mechanisms underlying fever. The fever is a physiological response regulated by the hypothalamus to stimuli of various kinds; thus, treatment of fever does not change the course of the causative disease or prevent the onset of febrile seizure [70]. Therefore, the pediatrician should advise parents to monitor hydration status and recommend antipyretics under certain conditions, such as if the child is in pain, informing about the most appropriate molecule, dose, and routes of administration. It is worth remembering that the use of aspirin should be avoided as it is potentially causative of pediatric Reye’s Syndrome [4]. A reasonable approach to the child with FUO should include discontinuation of nonessential medications; in cases of polypharmacy, medications should be discontinued individually, if possible. Empirical therapy may mask or delay the diagnosis of relevant pathological conditions. Therefore, if a child is in good clinical condition, the routine use of empiric therapy (antibiotic or anti-inflammatory) is generally not recommended. Similarly, in autoimmune diseases, steroid therapy can be initiated only after diagnostic confirmation and exclusion of oncological disease. Anyway, a multifaceted approach to the diagnostic workup of patients with FUO is necessary for both patients and their families to avoid an unnecessary diagnostic odyssey. Several specialists may be involved in the investigations’ decision process.

Children with FUO generally have a better prognosis than adults [36]. The outcome is related to the primary disease and is mostly favorable because children with FUO have a curable or self-limiting disease. In an earlier published series, 88% of children with FUO due to infection resolved completely, while 90% of those with inflammatory disease had chronic sequelae [9]. Even the most recent literature confirms that in most cases, the fever resolves or a specific diagnosis is reached [16]. In a large Chinese cohort, the resolution of fever in the absence of a diagnosis was reported in 49% and only 6.5% of all cases had persisting fever [30].

However, the evolution of FUO in children is not always benign: in the two historical series of the 1970s, the mortality was 6% and 9%, respectively [8,9], although it has lowered in more recent reports [16]. However, data from the literature regarding the prognosis of patients with FUO in whom the diagnosis has not been reached are discordant. Steele et al. followed 63 patients without an etiologic diagnosis over time: of these, nine presented a new episode of FUO but eventually, all recovered definitively [11]. In contrast, Miller et al. reported neurological sequelae in 8/29 patients with periodic fevers of unknown cause [43]. Many patients without a definitive diagnosis still have a good outcome, even if fever recurs [43,71]. In the long-term follow-up of 19 children with FUO without etiological diagnosis with a duration of more than 2 weeks, 16 recovered completely, two were subsequently diagnosed with sJIA and the remaining one had two subsequent episodes of intussusception [71]. In another series of 40 children with FUO without etiology lasting more than one month, who were evaluated by a pediatric rheumatologist, 37 had prolonged follow-up, and, of these, two developed IBD. In both children, the fever resolved after starting specific therapy [43].

## 7. Discussion

Fever of unknown origin is a relatively common pediatric condition that continues to be a challenging evaluation for pediatric diagnosticians. This is especially true in academic and tertiary referral care centers. Furthermore, in pediatric patients, on the one hand, it causes considerable anxiety to the parents; on the other hand, it may represent a source of confusion and frustration for the attending physician, leading to unnecessary and additional over-the-counter laboratory tests and medications (including antimicrobial agents).

Indeed, FUO is a challenge because it is often difficult to clinically distinguish between benign and life-threatening causes. The goal of the pediatrician is not to miss the diagnosis of both serious diseases and benign conditions, a cause of increased morbidity, if not timely treated.

In approaching this issue, the pediatrician must pay close attention to the child’s history and examination, nowadays aided by the development of molecular diagnostic tests, to differentiate infections from other causes and to distinguish serious, even lethal, conditions from benign ones. Gathering as much information as possible during the first visit is pivotal. After obtaining a detailed medical history, a thorough medical examination should be performed and repeated. As already stressed, an evaluation of the general appearance of the child and the presence or absence of “red flags” are crucial in deciding the setting and the intensity of further investigations. Moreover, specific signs and symptoms could suggest possible diagnoses and guide the workup. A well-designed, tailored laboratory workup protocol conducted according to local epidemiological information may save time and reduce unnecessary medical costs and disease sequelae. From the evaluation of simple CBCs and inflammatory biomarkers, although non-specific, we can have some suggestions: very high values of WBC, CRP, and ESR suggest autoimmune diseases but also infections; neither neoplastic disorders could be excluded, although such values may be mildly altered or even normal. Certainly, new, early markers able to distinguish between infectious and noninfectious forms would be desirable.

Special categories of children (with complex congenital heart disease, immunocompromised, CVC carriers, or coming from developing countries) deserve special consideration for being at higher risk for infectious diseases.

Despite the fact that the diagnostic algorithm of FUO in immunocompromised patients is largely like those of FUO in immunocompetent children, some issues need to be considered. Infectious causes of FUO in immunocompromised children are heterogeneous and include opportunistic pathogens [72]. Moreover, almost none of the microbials isolated from blood or other biological tissues can be considered a contaminant, and, thereby, specific therapies required when such pathogens are encountered. Furthermore, in this particularly vulnerable population, the compelling need to quickly identify the agent allows for an accelerated step up towards second-level tests or invasive procedures. Indeed, some first-level imaging findings rely on the host’s immune response (i.e., chest X-ray pulmonary consolidations in pneumonia), and, thus, they can be falsely negative in patients with attenuated immune responses. Less common causes of fever must not be forgotten. Lastly, FUO can be caused by specific conditions, such as immune reconstitution syndrome, which can occur in patients with AIDS who are on effective antiretroviral therapy.

Sometime the first-level exams allow us to exclude the most common and/or serious causes of FUO and indicate whether to proceed with further tests. The subsequent investigations should be targeted towards the diagnostic hypothesis and, consequently, blood tests and imaging should be wisely planned. Often, after an initial phase, the correct framework involves a multidisciplinary approach (infectious disease specialist, rheumatologist, or oncologist). Imaging should be carefully evaluated, considering the diagnostic suspicion and cost/benefit, too. Only after performing negative or doubtful first-level imaging and altered but inconclusive blood chemistry investigations, WB-MRI and scintigraphy should be planned. Such recently introduced investigations (WB-MRI and ^18^FGD-PET/CT) seem to be useful in certain contexts to identify infectious foci or neoplastic processes not clinically evident; however, specific studies in pediatric age are still limited.

Although the prognosis of FUO is favorable in most cases, it should be highlighted that its cause is not always benign. The pediatrician must pay close attention to the past and recent history and physical examination, aided by the old laboratory findings, the new molecular diagnostic tests, and (hopefully) new specific biomarkers to distinguish infections from other causes.

## 8. Future Perspectives

As the differential diagnosis in children with FUO is challenging, especially differentiating systemic-onset JIA (sJIA) from infectious diseases, new biomarkers are needed that support such a diagnostic workup. Recently, the measurements of serum myeloid-related protein 8/14, named MRP8/14, have been validated as a helpful tool supporting the diagnosis of sJIA in febrile children [73].

Moreover, serum calprotectin analyses are a helpful tool supporting the diagnosis of SJIA in children with prolonged fever or inflammatory disease. The detection of serum calprotectin by an immuno-turbidimetric assay might be implemented in clinical laboratory settings to facilitate its use as a diagnostic routine test in clinical practice [74].

Furthermore, advanced genetic testing is now available for this purpose. Next-generation metagenomic sequencing (mNGS) is a novel nucleic acid method for the detection of unknown and difficult pathogenic microorganisms, and its application has recently been introduced in the etiological diagnosis of FUO, although less reported. New studies have extended its application in identifying tumors in body fluids and cerebrospinal fluids. Therefore, mNGS could be a useful tool to rapidly detect both pathogens and neoplasms in children with FUO [75]. The early detection of mNGS can shorten both the time to reach diagnosis and the days of hospitalization and reduce unnecessary antibiotic consumption [76].

## Figures and Tables

**Table 1:** Etiologies of FUO in children.

Infectious Causes		Noninfectious Causes
Bacterial		Neoplastic Causes
Localized	Abscesses	Leukemia
	Mastoiditis	Lymphoma
	Sinusitis	Wilms tumor
	Osteomyelitis	Neuroblastoma
	Chronic meningitis/encephalitis	Pheochromocytoma
	Pyelonephritis/urinary infection	Soft-tissue sarcoma
	Pneumonia/empyema	Hepatocarcinoma
	Pericarditis	Langerhans cell histiocytosis
	Endocarditis	Hemophagocytic lymphohistiocytosis
	Mediastinitis	Macrophage activation syndrome
Systemic	*Bartonella henselae*	Autoimmune/Inflammatory
	*Staphylococcus* spp.	Juvenile idiopathic arthritis
	*Streptococcus* A and B	Systemic lupus erythematosus
	*Pseudomonas*	Kawasaki disease
	*Streptococcus pneumoniae*	Polyarteritis nodosa
	*Hemophilus influenzae*	Behçet disease
	*Brucella*	Rheumatic fever
	*Fusobacterium*	Wegener granulomatosis
	*Kingella kingae*	Sarcoidosis
	Mycobacteria	Antiphospholipid syndrome
	*Mycoplasma pneumoniae*	Takayasu arteritis
	*Francisella tularensis*	Ankylosing spondylitis
	*Coxiella burneti*	Chronic noninfectious osteomyelitis
	*Salmonella* sp.	Inflammatory bowel disease
	*Enterococcus* spp.	Thyroiditis
	*Borrelia burgdoferi*	
Viral		Miscellaneous
Cytomegalovirus (CMV)		Drug fever
Epstein Barr Virus (EBV)		Diabetes insipidus
Human immunodeficiency virus (HIV)		Familial dysautonomia
Parvovirus B-19 (HPV-B19)		Autoinflammatory diseases
Adenovirus		Kikuchi–Fujimoto disease
Herpes virus (HSV)		Castleman disease
Enterovirus		Sick-serum disease
Hepatitis virus A, B, C, E		Sweet syndrome
Influenza virus A and B		Ectodermal dysplasia
SARS-CoV-2		Pancreatitis
Arbovirus		Pediatric multisystem inflammatory syndrome
Fungal		Central nervous system dysfunction
Blastomycosis		Immunodeficiencies
Cryptococcosis		Fictitious fever
Histoplasmosis		
Candidiasis		
Aspergillosis		
Coccidioidomycosis		
Parasitic		
Leishmania		
Toxoplasma		
Malaria		
Amoeba		
Giardia		

**Table 2:** Anamnestic data associated with pathologic conditions.

Family history	Familial disease	Familial dysautonomia
	Brothers/sisters (age)	Viral infections
Physiological history	Ethnicity	Familial Mediterranean Fever
	Adoption	TB, HIV, hepatitis B and/or C, malaria, typhoid fever
	Kindergarten attendance	Viral infections, bacterial infections, *Kingella kingae*
	Food habits	Unpasteurized milk: TB, Brucella, Listeria, *E. coli*, *Campylobacter* Goat’s milk: Brucella, Salmonella, Listeriawater (well): Salmonella, Giardia, *Campylobacter* Undercooked meat: toxoplasma Picacism: Toxocara
	Vaccinations	Absence: invasive infections
	Sexual activity	Sexually transmitted infections, pelvic disease
	Piercings and tattoos	HIV, hepatitis B and/or C, infectious endocarditis
Past medical history	Comorbidities	Diabetes, autoimmunity, nephrotic syndrome, etc.
	Supports/devices	Bacterial infections
	Drugs in chronic	Drug-induced fever
	Travel/bathing (rivers and lakes)	Malaria, hepatitis A and/or E, Dengue, Leptospira, TB, visceral
Recent history		leishmania, schistosomiasis, Lyme Disease, rickettsia, tularemia, Chikungunya, Vibrionaceae
	Animals/insect bites/ticks	Zoonoses
	At risk contacts	TB
	Recurrent infections	UTIs, pharyngotonsillitis, skin infections
	Surgical interventions	Abdominal or pelvic abscesses
	Drugs	Drug-induced fever
	Anorexia	Leukemia, lymphoma
	Weight loss	Leukemia, lymphoma, IBD, TB, HIV
	Itching, night sweats	Lymphoma
	Severe asthenia	EBV, leukemia, lymphoma, systemic JIA, infective endocarditis

**Table 3:** II level investigations oriented by diagnostic suspicion.

Suspected Infectious Disease	Specific Test
TB exposure, travel, immigration	Mantoux test and IGRA
Travel to countries with malaria endemics	Thick and thin drop smearPCR plasmodium, Malaric antigen detection
Asthenia, lymphopenia, high AST and ALT	EBV serology
Contact with cats, lymphadenopathy	Bartonella serology
Consumption of unpasteurized dairy products	Brucella, listeria, *E. coli* serology
Picacism, ingestion of contaminated food	Toxocara, toxoplasma serology
Raw/undercooked meat, animal contact	Toxoplasma, tularemia serology
Asthenia, sexual activity, cytopenia	HIV test
Travel, hepatosplenomegaly	Mantoux test, stool culture, malaria research
Osteoarticular swelling/pain	Synovial fluid cultures, X-ray, MRI
Abdominal pain (abscess or UTI)	Ultrasound, CT scan
Suspected inflammatory disease	
JIA	ANA, slit lamp, ultrasound abdomen
SLE	ANA, C3 and C4, anti-dsDNA
Kawasaki disease	EKG and Echocardiogram
IBD	ANCA, ASCA, calprotectin, fecal occult blood test, bowel US, GI endoscopy
Suspected neoplasm	
Leukemia, HLH	LDH, uricemia, peripheral smear, myeloaspirate
Lymphoma	Lymph node biopsy
Neuroblastoma	Urinary VMA
Nonlocalized malignancy	Scintigraphy, WB-MRI
Wilms tumor	Abdomen ultrasound, CT scan of the abdomen

## Data Availability

No new data were created or analyzed in this study. Data sharing is not applicable to this article.

## Supplementary Materials

The following supporting information can be downloaded at https://www.mdpi.com/article/10.3390/children11010020/s1, Table S1: Case series of FUO in children published over 50 years; Table S2: Physical examination findings and possible diagnoses of FUO.
