# Supplementary material for: Update on Fever of Unknown Origin in Children: Focus on Etiologies and Clinical Approach
Source: Children (Basel). 2023 Dec 24;11(1):20. doi: 10.3390/children11010020 (PMC10814770; doi:10.3390/children11010020)
Supplement: Supplementary file 1 [file children-11-00020-s001.zip › children-2778350-supplementary.pdf]

Table S1: case series of FUO in children published over the last 50 years

Author (year)	Country	Study design	Total cases	Infections	Inflammatory	Neoplasiae	Miscellaneous	NO diagnosis
Hu B (2022)	Cina	Single center retrospective	1288	50.9%	4.9%	6.7%	26.6%	10.9%
Xu J (2022)	Cina	Single center retrospective	357	45.7%	37.2%	5.9%	11.2%	
Szymanski AM (2020)	USA	Single center retrospective	110	37.2%	14.5%	4.5%	5.4%	38.2%
Antoon (2018)	USA	Single center retrospective	102	41.2%	27.5%	17.7%	13.7%	
Chien YL (2017)	Cina	Single center prospective	93	37%	14%	17.2%	16%	15.1%
Kim JS (2017)	Corea	Single center retrospective	100	19%	15%	7%	8%	43%
Cho LY (2017)	Taiwan	Single center retrospective	126	27%	12.7	16.6%	19.8 (KD)	23.8%
Hassan RH (2014)	Egitto	Single center retrospective	127	36.2%	10.2%	7.9%	29.9%	15.7%
Mahmoudi S (2014)	Iran	Single center retrospective	95	26.6%	14.7%	7.4%	51.6%	
Tezer H (2012)	Turchia	Single center	77	50.7%	7.2%	14.4%	27.5%	10.3%
Chow A (2011)		Systematic Review	1638	51%	9%	6%	11%	23%
Joshi N (2008)	India	Single center retrospective	49	69%	2%	12%	4%	12%
Pasic S (2006)	Serbia	Single center prospective	185	37.5%	19.3%	6.4%	8.1%	30%
Chouchane S (2004)	Tunisia	Single center retrospective	67	56.7%	20.9%	3%	19.4%	
Ciftçi E (2003)	Turchia	Single center retrospective	102	44.2%	6.8%	11.7%	24.3%	12.8%
Cogulu O (2003)	Turchia	Single center retrospective	80	58.7%	6.2%	2.5%	20%	12.5%
Chantada G (1994)	Argentina	Single center retrospective	113	36.3%	13.3%	9.7%	21.2%	19.5%
Steele RW (1991)	USA	Single center retrospective	109	22%	6%	2%	3%	73%
Lohr JA (1977)	USA	Single center retrospective	54	33%	20%	13%	15%	19%
Pizzo PA (1975)	USA	Single center retrospective	100	52%	20%	6%	10%	12%
McClung HJ (1972)	USA	Single center retrospective	99	29.3%	11.1%	8.1%	19.1%	32.3%

**Table S2: Physical examination findings and possible diagnoses of FUO**		
SYSTEMIC	Anorexia<br>Weight/height<br>HR	Weight loss: leukemia, lymphoma, TB, chronic illness<br>Bradycardia: typhoid fever, malaria, leptospira, central fever/ drug fever
ABDOMEN	Hepatomegaly	Leukemia, lymphoma, granulomatous hepatitis, hemophagocytic lymphohistiocytosis, typhoid fever, viral hepatitis, salmonellosis, brucellosis, bartonellosis, Malaria, *Coxiella*, histoplasmosis
	Splenomegaly	Leukemia, lymphoma, TB, brucellosis, EBV, CMV, psittacosis, typhoid fever, HLH, Kikuchi-Fuijmoto disease
	Palpable mass	Neoplasms, abdominal abscesses
HEART	Heart murmur	Infective endocarditis, atrial myxoma
	Arrhythmias<br>Friction rubs	Rheumatic Fever, Lyme disease, myocardial abscesses<br>Infectious pericarditis or pericardial effusions (SLE)
LYMPH NODES	Lymphadenomegaly	Leukemia, lymphoma, bartonellosis, tuberculosis, lymphogranuloma venereum, CMV, EBV, adenovirus, HIV, toxoplasmosis, brucellosis, tularemia, mycobacteriosis, JIA, Kikuchi-Fuijmoto disease
MUSCULOSKELETAL	Bone pain	Osteomyelitis, neoplasms, infantile cortical hyperostosis
	Arthralgia/arthritis	JIA, Familial Mediterranean Fever, SLE, Lyme disease, brucellosis, lymphogranuloma venereum, Hyper-IgD syndrome
	Myalgias	Pyomyositis, viral myositis, brucellosis, trichinellosis, arbovirus infection, polyarteritis, subdiaphragmatic abscess
	Spinal pain	Discitis, spondylodiscitis, brucellosis, typhoid fever, infective endocarditis
GENITO-URINARY	Epididymitis	TB, lymphoma, brucellosis, leptospirosis, EBV, blastomycosis
SKIN AND APPENDAGES	Erythema nodosum	Infections, JIA, SLE, IBD, neoplasm
	Erythema migrans	Lyme disease
	Malar rash	SLE, Parvovirus
	Salmon rash	JIA
	Palpable purpura	PAN, Endocarditis, bacteremia, viral infections, rickettsia, vasculitis
	Seborrheic rash	Histiocytosis
	Digital clubbing	Heart disease, chronic pulmonary disease, hepatitis, CF, endocarditis
	Bullous lesions	Staphylococcus aureus infection
ORAL CAVITY PHARYNX	Pharyngeal hyperemia	CMV, EBV, toxoplasmosis, tularemia, leptospirosis
	Ulcers/aphthae	Behçet disease, SLE
	Gingival hypertrophy	Leukemia, Histiocytosis
	Epistaxis	Leukemia, psittacosis, rheumatic fever
	Dental pain or pharyngeal asymmetry	Dental abscess, sinusitis<br>Parapharyngeal abscesses, Lemierre syndrome
FACE	Pain, swelling	Mastoiditis, sinusitis
NECK	Meningism	Chronic meningitis<br>Mastoiditis, sinusitis, vertebral osteomyelitis
	Palpable mass	Lymphadenopathies, Lemierre syndrome, KD, suppurative thyroiditis
EYE	Ocular fundus altered	Miliar TB, toxoplasmosis, vasculitis
	Conjunctival hyperemia	EBV, leptospirosis, KD, tuberculosis, SLE, bartonellosis, Chlamydia
	Dry eye	Familial dysautonomy, SLE, PAN, Sjögren syndrome
	Uveitis	Tuberculosis, JIA, toxoplasmosis, sarcoidosis, SLE
	Retinal ischemia	PAN
	Opsoclonus/myoclonus	Neuroblastoma
